# Pharmacologic Approach to Defective Protein Trafficking in the E637K-hERG Mutant with PD-118057 and Thapsigargin

**DOI:** 10.1371/journal.pone.0065481

**Published:** 2013-06-19

**Authors:** Haiyan Mao, Xiaoli Lu, Justin Michael Karush, Xiaoyan Huang, Xi Yang, Yanna Ba, Ying Wang, Ningsheng Liu, Jianqing Zhou, Jiangfang Lian

**Affiliations:** 1 LiHuiLi Hospital, Medical School of NingBo University, NingBo, China; 2 People’s Hospital of Anji County, HuZhou, China; 3 Department of Thoracic Surgery, Memorial Sloan-Kettering Cancer Center, New York, New York, United States of America; 4 Department of Pathology, Key Laboratory of Antibody Technique of Ministry of Health, Nanjing Medical University; Nanjing, China; University of Bologna & Italian Institute of Technology, Italy

## Abstract

**Background:**

Treatment of LQT2 is inadequate. Many drugs which can pharmacologically rescue defective protein trafficking in LQT2 also result in potent blockade of HERG current, negating their therapeutic benefit. It is reported that PD-118057 and thapsigargin can rescue LQT2 without hERG channel blockade, but the precise mechanism of action is unknown. Furthermore, the effect of PD-118057 and thapsigargin on the dominant negative E637K-hERG mutant has not been previously investigated.

**Objective:**

In this study, we investigated: (a) the effect of PD-118057 and thapsigargin on the current amplitudes of WT-hERG and WT/E637K-hERG channels; (b) the effect of PD-118057 and thapsigargin on the biophysical properties of WT-hERG and WT/E637K-hERG channels; (c) whether drug treatment can rescue channel processing and trafficking defects of the WT/E637K-hERG mutant.

**Methods:**

The whole-cell Patch-clamp technique was used to assess the effect of PD-118057 and thapsigargin on the electrophysiological characteristics of the rapidly activating delayed rectifier K^+^ current (I_kr_) of the hERG protein channel. Western blot was done to investigate pharmacological rescue on hERG protein channel function.

**Results:**

In our study, PD-118057 was shown to significantly enhance both the maximum current amplitude and tail current amplitude, but did not alter the gating and kinetic properties of the WT-hERG channel, with the exception of accelerating steady-state inactivation. Additionally, thapsigargin shows a similar result as PD-118057 for the WT-hERG channel, but with the exception of attenuating steady-state inactivation. However, for the WT/E637K-hERG channel, PD-118057 had no effect on either the current or on the gating and kinetic properties. Furthermore, thapsigargin treatment did not alter the current or the gating and kinetic properties of the WT/E637K-hERG channel, with the exception of opening at more positive voltages.

**Conclusion:**

Our findings illustrate that neither PD-118057 nor thapsigargin play a role in correcting the dominant-negative effect of the E637K-hERG mutant.

## Introduction

Mutation in the human ether-a-go-go-related gene (hERG or KCNH2) is responsible for reducing the rapidly activating component of the delayed rectifier potassium current (I_Kr_), which can prolong the QT interval and induce arrhythmia (Type 2 Long QT syndrome, LQT2) [1]. To date, genetic analyses have identified approximately 300 LQT2-associated KCNH2 mutations, which represent the most common mechanism of hERG channel dysfunction. Mutations in hERG lead to reduced expression of functional surface membrane channels as well as lower current magnitudes. Due to defective protein-trafficking, mutant channels are retained in the endoplasmic reticulum (ER) and fail to reach the plasma membrane [2], [3], [4], [5], [6]. It has been shown that correction of protein folding defects by pharmacologic chaperones (hERG channel blockers) can restore proper protein-trafficking [6]. However, their affinity for hERG channel blockade leads to acquired Long QT syndrome (LQTS) and severely limits their efficacy. This is due to binding of amino acid residues within the inner portion of the 6th transmembrane segment (S6) of hERG channel proteins [7], [8], [9]. Numerous types of drugs, with a wide range of chemical structures, are prone to hERG channel inhibition [10], [11]. Many drugs have been removed from the market because of the potential lethality of hERG channel blockade, and screening for hERG block activity has become an important part of modern drug development [12]. As such, there is great interest in developing compounds that correct trafficking defects without hERG channel blockade.

Several drugs, classified as small molecule activators of hERG channels, have recently been reported which appear to rescue I_Kr_ channels without significant hERG blockade, including RPR260243, NS1643, and PD-118057 (Table S1). Of these drugs, we tested PD-118057 because it has the most efficient current-enhancing effect on hERG current, without affecting the voltage dependence and kinetics of gating parameters, nor does it require open conformation of the hERG channel [13]–[15]. PD-118057 represents one of the two major classes of hERG channel activators, but its precise mechanism of action is unknown, and safety and efficacy issues have yet to be adequately addressed. Unlike NS1643, whose activator effect is known to be potentiated by a mutated aromatic residue (Phe656) on S6 [14], no data exists regarding the effect of mutation on PD-118057.

Additionally, thapsigargin is a compound which inhibits endoplasmic reticulum Ca+-ATPase activity and has been shown to correct trafficking defects without hERG blockade [16]. We chose to assess thapsigargin because it promotes the relocation of intracellular proteins through its effect on Ca+ dependent molecular chaperone activity, not through binding to the hERG channel itself. Thapsigargin, which is classified as a sesquiterpene lactone, can selectively rescue several different LQT2 mutations, including the C terminus mutation G601S and F805C, but not N470D [16]. The E637K-hERG mutant, which substitutes lysine for glutamic acid at position 637 in the pore-S6 loop transmembrane segment of hERG, has been identified as a dominant-negative mutation [14]. We undertook the present study to investigate: (a) the effect of PD-118057 and thapsigargin on the current amplitudes of WT-hERG and WT/E637K-hERG channels; (b) the effect of PD-118057 and thapsigargin on the biophysical properties of WT-hERG and WT/E637K-hERG channels; (c) whether drug treatment can rescue channel processing and trafficking defects of the WT/E637K-hERG mutant.

## Materials and Methods

### Plasmid Construction

HERG cDNA was subcloned into the pcDNA3 vector (Invitrogen,California, USA) at BamHI/EcoRI restriction sites. Mutant pcDNA3 E637K-hERG construct was made by overlap extension PCR and verified by DNA sequencing. The primer sequences were as follows: outer primers 5′-gccacgccagcaccggggccatgc-3′ (forward) and 5′-gtgtggtcttgaacttcatggccagggc-3′ (backward); mutagenesis primers 5′-caacaccaactcaaagaagatcttctcc-3′ (forward) and 5′-ggagaagatcttctttgagttggtgttg-3′ (backward). Plasmid DNA for mammalian expression was amplified in Escherichia coli TOP10 competent cells.

### Cell Lines and Drug Exposure

Human embryonic kidney 293 (HEK293) cells were cultured in Dulbecco’s Modified Eagle Medium (DMEM, Thermo scientific) supplemented with 10% fetal bovine serum in 5% CO2 incubator at 37°C. HEK293 cells were transiently transfected with 3.2 µg of WT-hERG and/or 3.2 µg of E637K-hERG plasmids using Lipofectamine™ 2000 according to the manufacturer’s instruction (Invitrogen, California, USA). 0.8 µg of pRK5-GFP plasmid was co-transfected to monitor transfection efficacy. Thapsigargin (Sigma, St. Louis, MO, USA; 1 mmol/L stock dissolved in DMSO), PD-118057 (Sigma, St. Louis, MO, USA; 5 mmol/L stock dissolved in DMSO) were added to the culture media for different time periods before analyzing. Final DMSO concentrations in medium was <0.1%.Incubating HEK293 cells expressing WT-hERG, WT/E637K-hERG or E637K-hERG overnight in 0.1% DMSO had no effect on I_hERG_ or complex glycosylation.

### Whole-cell Patch-Clamp Recordings

To examine the effect of drug treatment on WT/E637K-hERG by patch clamp technique, HEK293 cells were harvested at 24 (DNA plasmid only) or 48 hours (DNA plasmid followed by drugs) and superfused with 4-(2-hydroxyethyl)-1-piperazine ethane sulphonic acid (HEPES)-buffered Tyrode solution containing 137 mM NaCl, 4 mM KCl, 1.8 mM CaCl_2_, 1 mM MgCl_2_, 10 mM glucose, and 10 mM HEPES (pH 7.4 adjusted with NaOH). The internal pipette solution contained 130 mM KCl, 1 mM MgCl_2_, 5 mM [Ethylenebis(oxyethlenenitrilo)]-Tetraacetic Acid (EGTA), 5 mM Mg-ATP, and 10 mM HEPES (pH 7.2 adjusted with KOH) as previously described [15], [16], [17], [18]. Membrane currents were recorded in whole-cell configuration using pipettes with a tip resistance of 2 to 5 Mohms when filled with the internal solution. The electrodes were connected to an Axopatch 700A amplifier (Axon Instruments,California, USA) and digitized at 2 kHz with an analogue-to-digital converter (DigiData 1200B; Axon Instruments, California, USA). All experiments were done at room temperature.

### Western Blot Analysis

Western blot analysis of hERG protein was performed as previously described [19]. Briefly, HEK293 cells were solubilized in ice-cold Radio-Immunoprecipitation Assay (RIPA) buffer with freshly added protease inhibitors and phenylmethanesulfony fluoride (PMSF) (Solarbio,Beijing,China). Proteins were separated on 8% SDS-polyacrylamide gels and transferred to polyvinylidene difluoride (PVDF) membranes. Membranes were blocked for 2-hrs with blocking solution (5% nonfat dry milk powder and 0.1%Tween −20 in TBS) and subsequently incubated with rabbit polyclonal anti-hERG antibody (Alomone Labs,Jerusalem, Israel) at 4°C over night, followed by alkaline phosphatase goat anti-rabbit IgG (ZSGB-BIO, Beijing, China) for 2-hrs at room temperature. Protein bands were detected with the alkaline phosphatase substrate BCIP/NBT colorimetric determination kit (ZSGB-BIO, Beijing, China).

### Statistical Analysis

The pCLAMP Ver. 9.2 software (Axon Instruments, California, USA) was used to generate voltage clamp protocols, acquire data and analyze current traces. Voltage-dependence activation was determined by fitting the peak tail current (I_tail_) with Boltzmann function: I/Imax = 1−{(1+ exp[(Vt−V_1/2_)/k]}^−1^. V_1/2_ represents the half-maximal voltage and k is the slope factor. Time constants of inactivation or recovery from inactivation were fitted by single exponential standard. Fast and slow time constants of deactivation were fitted by double exponential standard. Results were expressed as means ± SE, and statistical analyses were performed with Student’s t-test. Results were considered to be statistically significant when *P*<0.05.

## Results

### Effect of PD-118057 and Thapsigargin on WT-hERG and WT/E637K-hERG Current Amplitudes

**Figure 1:**
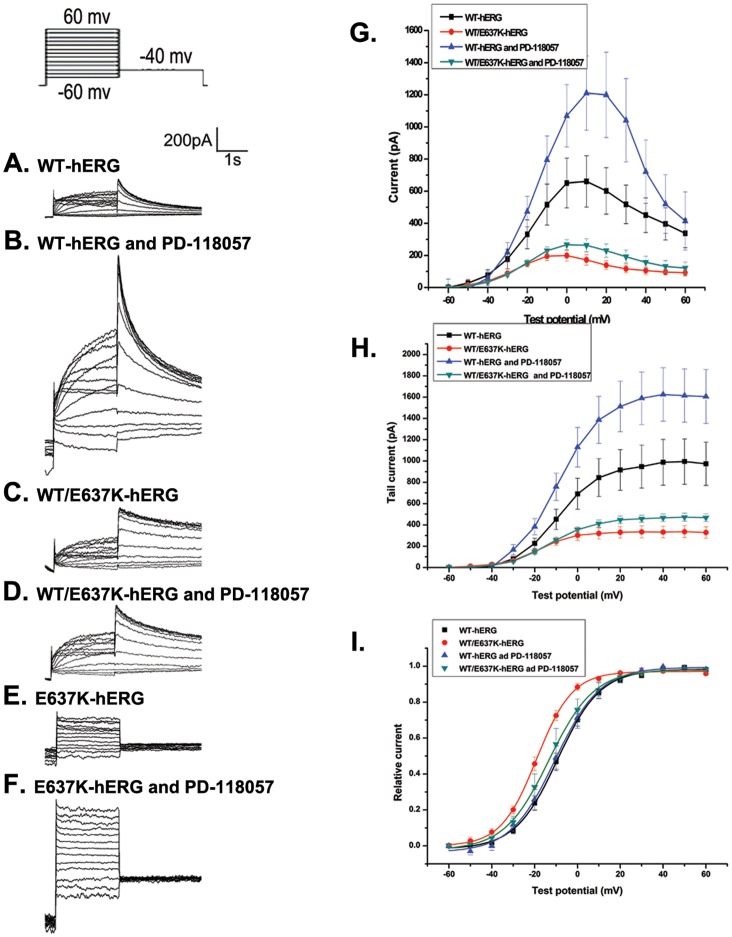
Effect of PD-118057 (3 µM) on voltage-dependent activation of hERG channel. Inset shows the voltage clamp protocol. a-f: Representative current traces in HEK293 cells transfected with WT-hERG, WT/E637K-hERG, and E637K-hERG in the presence or absence of PD-118057. g, h: Current-voltage (I-V) relationships for peak and tail current amplitudes of WT-hERG and WT/E637K-hERG transfected cells in the presence and absence of PD-118057. i: Amplitudes of tail currents of WT-hERG and WT/E637K-hERG channels in the presence or absence of PD-119057 are plotted as a function of the test potential and fitted to a Boltzmann function (n = 6).

**Figure 2:**
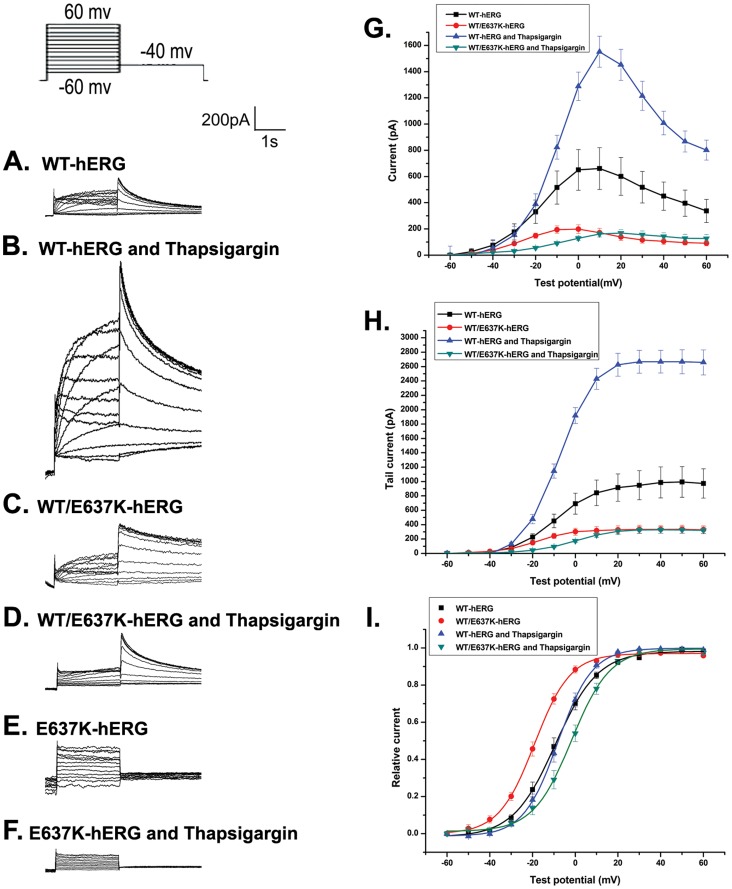
Effect of thapsigargin (1 µM) on voltage-dependent activation of hERG channel. Inset shows the voltage clamp protocol. a-f: Representative current traces in HEK293 cells transfected with WT-hERG, WT/E637K-hERG, and E637K-hERG in the presence or absence of thapsigargin. g, h: Current-voltage (I-V) relationships for peak and tail current amplitudes of WT-hERG and WT/E637K-hERG transfected cells in the presence or absence of thapsigargin. i: Amplitudes of tail currents of WT-hERG or WT/E637K-hERG channels in the presence or absence of thapsigargin are plotted as a function of the test potential and fitted to a Boltzmann function (n = 6).

We first measured the peak and tail currents in untreated HEK293 cells expressing WT-hERG or WT/E637K-hERG channels. Currents were elicited by voltage clamp protocol as previously described [20], [21]. Cells were depolarized to test voltages between −60 mV and 60 mV in 10 mV increments for 2 s from a −80 mV holding potential, followed by −40 mV for 4 s to elicit tail currents. Figure 1 shows representative whole cell currents and the corresponding current–voltage (I–V) relationship of the maximal and tail current amplitudes. As expected, untreated WT/E637K-hERG cells demonstrated a significant reduction in maximal current amplitude compared to WT-hERG cells (198.66±33.39 pA versus 660.67±159.55 pA; n = 6, p<0.05, Fig. 1G and 2G). A similar reduction in tail current was observed in WT/E637K-hERG expressing cells compared to WT-hERG (333.12±1.48 pA versus 981.28±5.94 pA; n = 6, P<0.05, Fig. 1H and 2H), confirming the negative effect of WT/E637K-hERG expression.

Next, we treated HEK293 cells expressing WT-hERG or WT/E637K-hERG channels with either PD-118057 (3 µM) or thapsigargin (1 µM) and compared current amplitudes to untreated controls. Single drug concentrations were chosen due to previous optimization of these drugs in terms of both efficacy of hERG current rescue as well as prevention of pharmacologically induced QT prolongation [13], [17] In WT-hERG expressing cells, PD-118057 treatment resulted in a significant increase in both maximal current amplitude (1310.96±230.65 pA versus 660.67±159.55 pA control; n = 6, p<0.05, Fig. 1G) and tail current amplitude (1620.20±11.12 pA versus 981.28±5.94 pA control; n = 6, P<0.05, Fig. 1H), but failed to rescue either the maximal current amplitude (279.47±29.05 pA versus 198.66±33.39 pA control; n = 6, p>0.05, Fig. 1G) or the tail current amplitude (468.43±2.06 pA versus 333.12±1.48 pA control; n = 6, p>0.05) in cells expressing WT/E637K-hERG. Similarly, thapsigargin treatment also enhanced both the maximal current amplitude (1551.79±117.55pA versus 660.67±159.55 pA control; n = 6, p<0.05, Fig. 2G) and tail current amplitude (2680.42±10.65 pA versus 981.28±5.94 pA control; n = 6, p<0.05, Fig. 2H) in WT-hERG expressing cells but failed to rescue either the maximal current amplitude (169.18±25.00 pA versus 198.66±33.39 pA control; n = 6, p>0.05, Fig. 2G) or the tail current amplitude (329.84±2.63 pA versus 333.12±1.48 pA control; n = 6, p>0.05, Fig. 2H) in WT/E637K-hERG cells.

### Effect of PD-118057 and Thapsigargin on Gating Properties of WT-hERG and WT/E637K-hERG Protein Channels

To assess the effect of PD-118057 and thapsigargin on the voltage dependence of protein channel activation, we normalized the tail currents of cells expressing WT-hERG and WT/E637K-hERG before and after drug treatment. Currents were plotted as a function of the test potential and fitted to a Boltzmann function, where V_1/2_ is the half-maximum activation voltage and k is the slope factor representing the steepness of the voltage dependence. In cells expressing WT-hERG, PD-118057 treatment resulted in no significant change in either the activation voltage (V_1/2_ = −10.38±0.54 mV versus −8.66±0.31 mV control; n = 6, p>0.05; fig. 1I) or the slope factor (k = 10.49±0.31 mV versus 9.99±0.34 mV control; n = 6, p>0.05; fig. 1I), which is consistent with the observations by Zhou and colleagues [15]. Similarly, thapsigargin did not alter the activation voltage (V_1/2_ = −7.90±0.23 mV versus −8.66±0.31 mV control; n = 6, p>0.05; fig. 2I) or slope factor (k = 8.06±0.20 versus 9.99±0.43 mV control; n = 6, p>0.05; fig. 2I) in WT-hERG cells. In cells expressing WT/E637K-hERG, PD-118057 treatment also resulted in no difference in activation voltage (V_1/2_ = −13.26±0.34 mV versus −18.24±0.30 mV control; n = 6, p>0.05; fig. 1I) or slope factor (k = 10.45±0.27 mV versus 8.34±0.27 mV control; n = 6, p>0.05; fig. 1I) However, thapsigargin treatment did result in a significant change in activation voltage in WT/E637K-hERG expressing cells (V_1/2_ = −1.20±0.44 mV versus −18.24±0.30 mV control; n = 6, p<0.05, Fig. 2I) but no change in slope factor (k = 8.98±0.39 mV versus 8.34±0.27 mV control). These results indicate that WT/E637K-hERG channels open at more positive voltages after thapsigargin treatment.

#### Steady-state inactivation

**Figure 3:**
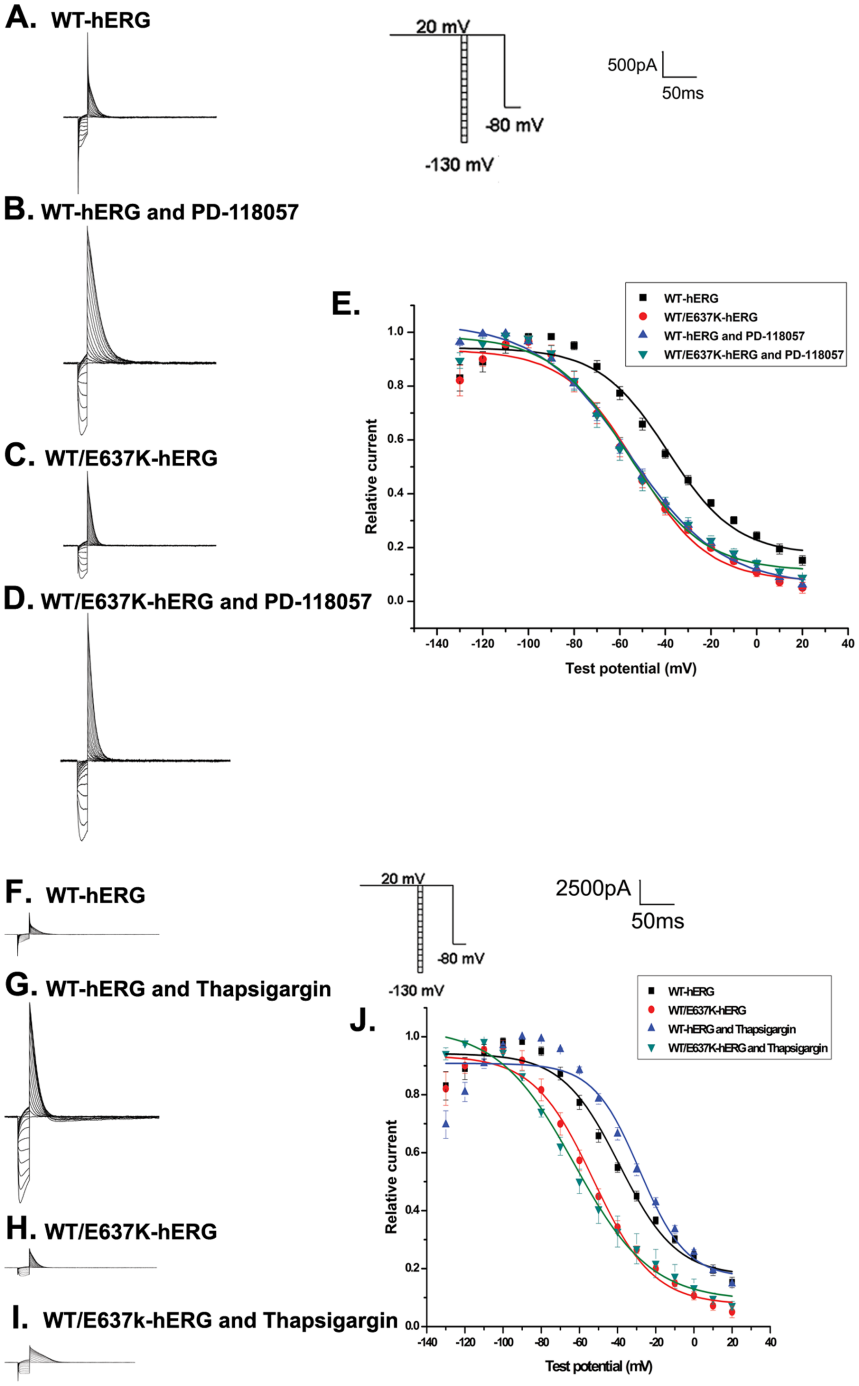
Effect of PD-118057 (3 µM) and thapsigargin (1 µM) on steady-state inactivation of hERG channel. Inset shows the voltage clamp protocol. a-d: Representative current traces in HEK293 cells transfected with WT-hERG or WT/E637K-hERG plasmids in the presence or absence of PD-118057 and thapsigargin. e: Normalized steady-state inactivation curves in cells transfected with WT-hERG or WT/E637K-hERG plasmids in the presence or absence of drug (n = 6).

To analyze the steady-state inactivation, test potentials between −130 mV and 20 mV in 10 mV increments for 20 ms were applied after a depolarizing pulse back to 20 mV for 4 s. This was followed by a test pulse to 20 mV for 500 ms before returning to a holding potential of −80 mV (Fig. 3, inset). Figures 3A–D and 3F–I show the representative voltage clamp recordings and figure 3E and 3J depict the normalized steady-state inactivation curves. In WT-hERG cells, PD-118057 treatment resulted in a significant change in steady-state inactivation (V_1/2_ = −55.83±1.42 mV versus −39.07±3.13 mV control, n = 6, p<0.05) but no change in slope factor (k = 20.20±1.52 mV versus 14.94±2.88 mV control). Thapsigargin treatment also resulted in an alteration in steady-state inactivation (V_1/2_ = −28.43±4.85 mV versus −39.07±3.13 mV control, n = 6, p<0.05) in WT-hERG cells but no change in slope factor (k = 12.19±4.21 mV versus 14.94±2.88 mV control). WT/E637K-hERG channels were largely unaffected by either PD-118057 (V_1/2_ = −58.69±1.77 mV, k = 16.64±2.13 mV) or thapsigargin treatment (V_1/2_ = −62.62±2.15 mV, k = 19.99±2.23 mV) compared to untreated control (V_1/2_ = −53.40±2.53 mV, k = 15.66±2.52 mV, n = 6, p>0.05). Our results demonstrate that both PD-118057 and thapsigargin treatment alter the steady-state inactivation kinetics of WT-hERG channels, but fail to affect WT/E637K-hERG channels.

#### Inactivation time course

**Figure 4:**
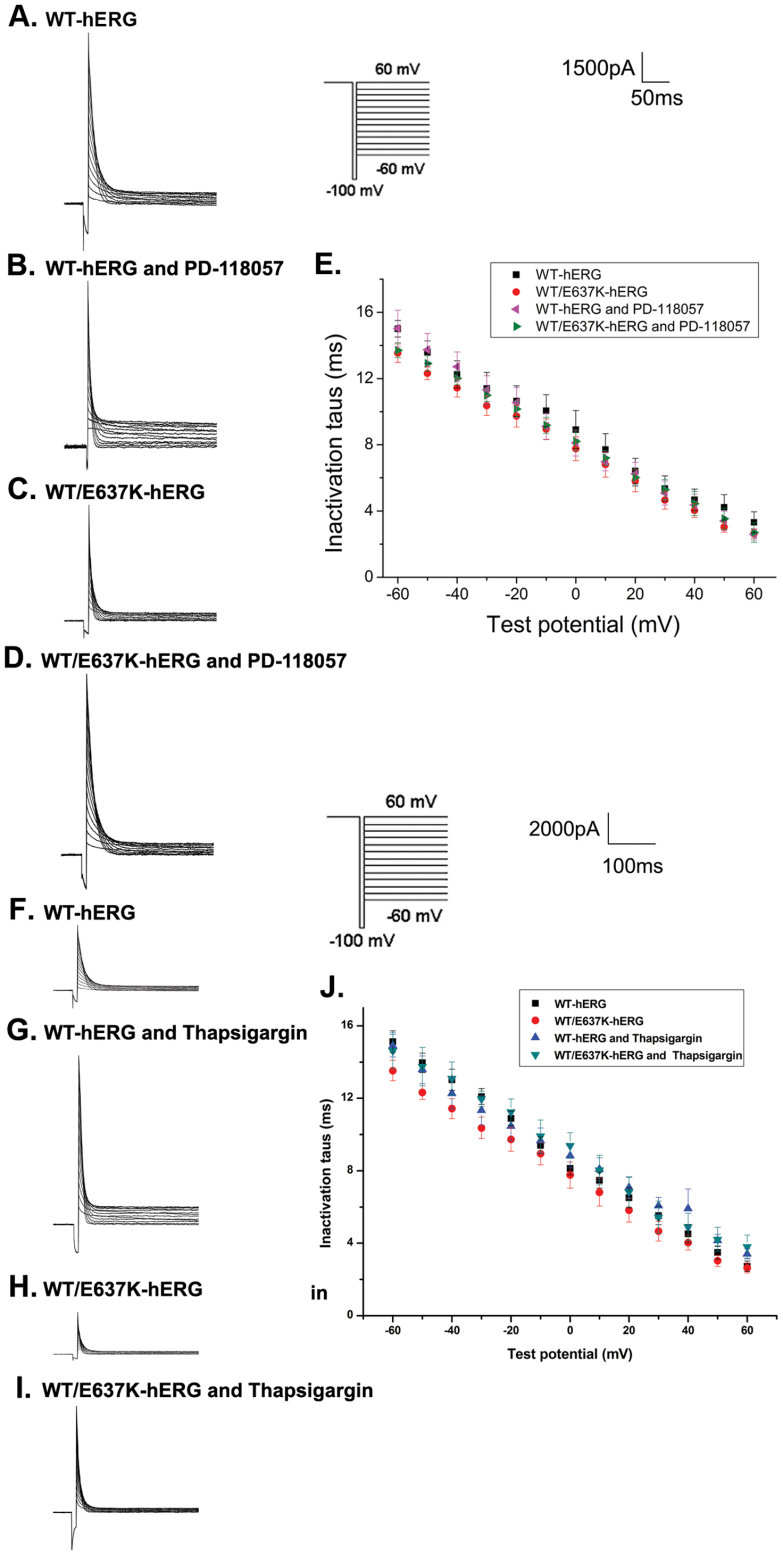
Effect of PD-118057 (3 µM) and thapsigargin (1 µM) on time courses of inactivation of hERG channel. Inset shows the voltage clamp protocol. a-d: Representative current traces of time courses and inactivation time constants (tau, τ) in HEK293 cells transfected with WT-hERG or WT-hERG/E637K-hERG plasmids in the presence or absence of PD-118057 and thapsigargin. e: τ was measured by fitting inactivation currents of WT-hERG or WT-hERG/E637K-hERG channel, in the presence or absence of PD-118057 and thapsigargin during test pulse at each potential, with a single exponential function (n = 6).

We next estimated the inactivation time course as previously described [18]. First, cells were depolarized from a holding potential of −80 mV to 60 mV for 2 s, hyperpolarized to −100 mV for 10 ms to allow hERG protein channels to recover from inactivation, and then depolarized to a test potential between −60 mV and 60 mV in 10 mV increments for 3 s to record the inactivation current (Fig. 4, inset). Figure 4A–D and 4F–I depict the typical current tracings of cells expressing WT-hERG and WT/E637K-hERG in the presence and absence of PD-118057 or thapsigargin. The time constants of inactivation were fitted as a single exponential function with the average (±SE) plotted in figure 4E and 4J. We found that exposure to PD-118057 or thapsigargin had no significant effect on the inactivation time course of either WT-hERG or WT/E637K-hERG channels (n = 6, P>0.05). These results demonstrate that neither PD-118057 nor thapsigargin affects the inactivation kinetics of WT-hERG or WT/E637K-hERG channels.

#### Recovery from inactivation

**Figure 5:**
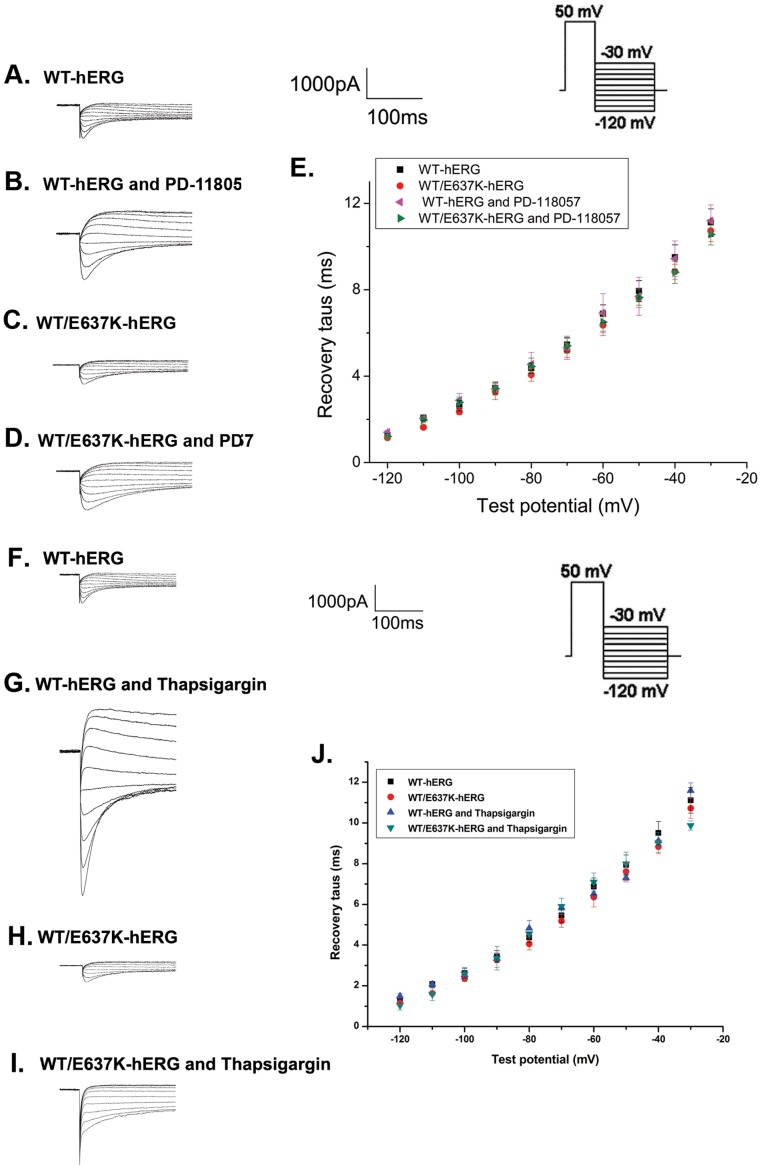
Effect of PD-118057 (3 µM) and thapsigargin (1 µM) on recovery from inactivation of hERG channel. Insert shows the voltage clamp protocol. a-d: Representative recovery from inactivation traces in HEK293 cells transfected with WT-hERG or WT/E637K-hERG plasmids in the presence or absence of PD-118057 and thapsigargin. e: Time constants (tau, τ) for hERG channel recovery from inactivation are plotted as a function of test voltages for WT-hERG or WT/E637K-hERG plasmids in the presence or absence of drug (n = 6).

To analyze the recovery from inactivation, cells were depolarized to 50 mV for 1.5 s and then repolarized to a test potential between −120 mV and −30 mV for 3 s to elicit tail currents (Fig. 5, inset). Figure 5A–D and 5F–I show the current tracings from cells expressing WT-hERG and WT/E637K-hERG channels in the presence and absence of PD-118057 or thapsigargin. Recovery from inactivation is represented by the initial phase of these currents, which were fitted as a single exponential function of the test potential to obtain the time constant of recovery from inactivation (Fig. 5E and 5J). As our data shows, the inactivation properties of cells expressing either WT-hERG or WT/E637K-hERG channels were not affected by PD-118057 or thapsigargin (n = 6, P>0.05).

#### Deactivation

**Figure 6:**
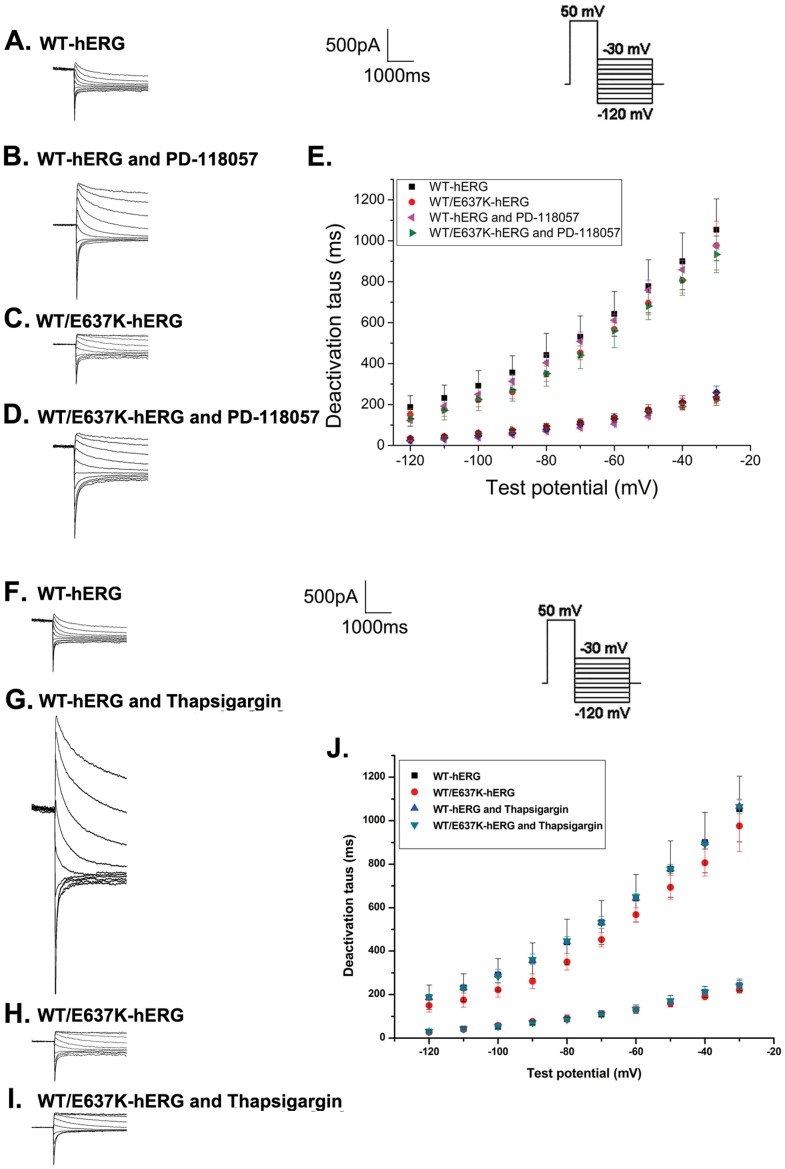
Effect of PD-118057 (3 µM) and thapsigargin (1 µM) on deactivation of hERG channel. Insert shows the voltage clamp protocol. a-d: Representative deactivation traces in HEK293 cells transfected with WT-hERG or WT/E637K-hERG in the presence or absence of PD-118057 and thapsigargin (arrow marks the deactivation phase). e: Fast and slow components of deactivation time constants (tau, τ) are plotted as a function of test potentials for WT-hERG or WT/E637K-hERG plasmids in the presence or absence of drug (n = 6).

Deactivation was measured by using the same protocol as recovery from inactivation (Fig. 6, inset). Figures 6A–D and 6F–I depict the typical current tracings from cells expressing WT-hERG and WT/E637K-hERG before and after exposure to PD-118057 or thapsigargin. The fast and slow deactivation time courses were measured by fitting deactivating tail currents during test pulses to a double exponential function. As the results show (Fig. 6E and 6J), no significant differences were found in the fast and slow time constants of deactivation for WT-hERG and WT/E637K-hERG channels treated by PD-118057 or thapsigargin compared to control (n = 6, P>0.05).

### Effect of PD-118057 and Thapsigargin on Channel Processing and Trafficking

**Figure 7:**
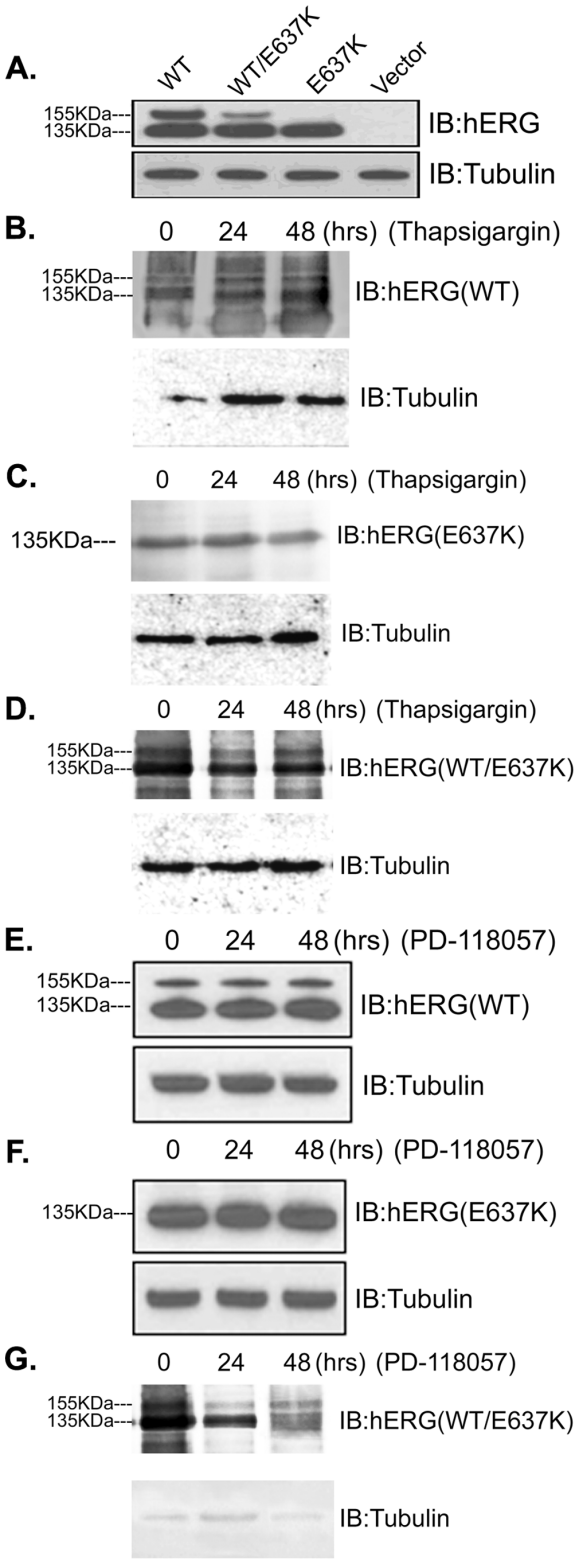
Analysis of hERG protein expression in HEK293 cells. a. Representative protein expression of untreated WT-hERG, WT/E637K-hERG, and E637K-hERG channels. b–d. Representative protein expression of WT-hERG, WT/E637K-hERG and E637K-hERG channels treated with 1 uM thapsigargin for 24 h to 48 h, respectively. Thapsigargin has no effect on the protein expression profile of either WT/E637K-hERG or E637K-hERG. e–f. Representative protein expression of WT-hERG, WT/E637K-hERG and E637K-hERG channels treated with 3 µM PD-118057 for 24 h to 48 h, respectively. As demonstrated, PD-118057 also has no effect on the protein expression profile of either WT/E637K-hERG or E637K-hERG channels.

To study whether PD-118057 and thapsigargin can pharmacologically rescue the dominant-negative effect of E637K-hERG channel function, hERG channel protein was analyzed by Western blot. HEK293 cells expressing WT-hERG, E637K-hERG and WT/E637K-hERG were incubated in PD-118057 or thapsigargin for 24 to 48 hours. As fig. 7A shows, HEK293 cells expressing WT-hERG undergo core and complex glycosylation during normal biogenesis, and the presence of two single protein bands at approximately 135 kDa and 155 kDa represents the hERG signature. The 155 kDa band represents the complexly glycosylated (mature) form while the lower band (135 kDa) represents the core-glycosylated (immature) form of the channel protein, which is retained in the ER [17], [19]. The appearance of a 155 kDa band is often used as an indicator of channel rescue [6], [7], [22]. E637K-hERG cells have an atypical hERG expression pattern with only a single band at 135 kDa. Moreover, cells expressing WT/E637K-hERG express two protein bands at approximately 135 kDa and 155 kDa, but the 155 kDa protein band is fainter than that of cells expressing WT-hERG. Fig. 7B–G show the Western blot analysis of cells expressing WT-hERG, E637K-hERG and WT/E637K-hERG protein channels before and after treatment with PD-118057 or thapsigargin. As expected with normal protein trafficking, WT-hERG expressing cells have both the 155 kDa and 135 kDa band, and PD-118057 and thapsigargin treatment had no effect on them at either 24 or 48 hours. In cells expressing E637K-hERG, treatment with either drug also did not result in the appearance of a 155 kDa band, consistent with failure to rescue. Similarly, cells expressing WT/E637K-hERG were unaffected by PD-118057 or thapsigargin treatment. These data suggest that neither PD-118057 nor thapsigargin can rescue the dominant- negative effect of E637K-hERG channel function.

## Discussion

To date, treatment of LQT2 is inadequate. Beta-blockers are reported to be only 59% effective in preventing cardiac events in patients with LQT2 [23], [24]. It has been suggested that drugs which activate cardiac K^+^ channels could be used to enhance net repolarizing currents, which are reduced by gene mutations as well as hERG channel blockade. HERG channel activators prevent arrhythmia by suppressing action potential duration alternans [25], [26]. PD-118057 is a novel hERG channel activator that does not cause hERG blockade, and is a potential therapeutic option for LQT2. Additionally, thapsigargin, an inhibitor of endoplasmic reticulum Ca^2+^-ATPase, has been shown to rescue LQT2-associated KCNH2 mutations without hERG channel blockade. Our study investigates the effect of PD-118057 and thapsigargin on the current amplitude and gating properties of hERG and WT/E637K-hERG channels, and whether drug treatment can rescue channel processing and trafficking defects of the E637K-hERG mutant, which has not been previously reported.

In our study, PD-118057 was shown to significantly enhance both the maximum current amplitude and tail current amplitude of WT-hERG cells. However, PD-118057 did not significantly alter the gating and kinetic properties of the WT-hERG channel, with the exception of accelerating steady-state inactivation. These results are consistent with a previous study which reported the activity of PD-118057 was voltage-dependent, enhanced the magnitude of hERG current, and did not significantly alter the gating and kinetic properties of the hERG channel [27]. In contrast to PD-118057, RPR260243 is a type I hERG agonist which enhances current magnitude by attenuating inactivation, as well as slowing hERG channel deactivation. Because the binding site of PD-118057 is a distant hydrophobic pocket formed by residues located on the pore helix, which is a nearby region of S6, PD-118057 does not directly affect deactivation [28]. Casis and colleagues [29] suggest that the decrease in rectification of hERG by PD-118057 indicates that it may have a similar mechanism as NS1643, which is another type 2 hERG agonist. NS1643 reduces the rectification of hERG through fast channel inactivation, thus slowing the onset of hERG inactivation. It is reported that a number of modulators positively regulate hERG channels, including phosphatidylinositol 4, 5-bisphosphate [30], PKC [31], direct binding of cAMP [32] and Polyamines [33]. However, their regulatory mechanisms often lack specificity, resulting in the functional alteration of other ion channels, and are typically associated with gating and/or kinetic changes. Although recent studies cannot rule out the possibility that PD-118057 acts through indirect mechanisms to increase hERG currents, our results showing the failure of PD-118057 to significantly affect the gating and kinetic properties of the hERG channel cause us to speculate that PD-118057 increases current amplitude by binding to the channel directly and increasing its open probability and activation potential.

Additionally, our study is the first to demonstrate that PD118057 is unable to rescue the dominant-negative suppression of the E637K-hERG mutant on WT-hERG channel function. This may be due to PD-118057 being a specific I_Kr_ agonist, and having no effect on I_Na_, I_Ca_, I_K1_, or I_Ks_ [27]. Given the requirement for PD-118057 to bind directly to the channel, the reduction of open I_Kr_ channel conformation seen with WT/E637K-hERG may prevent PD-118057 from rescuing its function. Future work might be performed to test this hypothesis. We also found that PD-118057 failed to enhance the current magnitude of E637K-hERG channel. Given the hypothesis that PD-118057 binds to a distant hydrophobic pocket formed by residues located on the pore helix and a nearby region of S6, mutation in this region may abolish current activation by PD-118057 [34]. Further studies might be performed to test this hypothesis, such as using PD-118057 to treat several different missense mutations located on the pore helix or nearby region of S6, allowing us to assess the specific binding site of PD-118057.

Our study also demonstrates that thapsigargin significantly enhances both the maximum current amplitude and tail current amplitude of WT-hERG cells. For cell expressing the WT-hERG channel, thapsigargin treatment increased the maximal current amplitudes by 57.43% compared to control cells. Similarly, the tail current amplitude was increased 63.39% after thapsigargin treatment. However, thapsigargin did not significantly alter the gating or kinetic properties of the WT-hERG channel, with the exception of attenuating steady-state inactivation. Moreover, our study is the first to demonstrate that thapsigargin fails to rescue the dominant-negative suppression of the E637K-hERG mutant on WT-hERG channel function. It is possible that because WT/E637K-hERG mutants consist of wild-type and E637K mutant channels, that such heteromultimers function as potassium channels but suppress normal channel function. Although thapsigargin significantly increased the WT-hERG channel current amplitude, it failed to rescue the E637K/WT-hERG current.

It is reported that dominant-negative mutation of hERG results in delayed myocellular repolarization and may promote L-type Ca^2+^ channel reactivation, leading to secondary depolarization and torsade de pointes arrhythmia [35]–[37]. Thapsigargin acts through inhibition of endoplasmic reticulum calcium ATPase, thus modulating the calcium-dependent chaperone proteins [13]. However, thapsigargin is unable to rescue all LQT2 mutations. It has been shown to rescue the G601S (S5/pore domain) and F805C (C-terminal junction) mutations, but not the N470D (S2 transmembrane domain) mutation [38]–[41]. Based on these reports, we hypothesized that thapsigargin targeted the region which is nearby the C-terminus. However, our study shows that thapsigargin fails to rescue trafficking defects of the E637K-hERG mutant, which is a novel missense mutation located in the pore-S6 loop transmembrane segment of hERG, nearby the C-terminus [14]. So in future work, we propose to investigate the molecular target for thapsigargin and whether thapsigargin can correct other LQT2 mutations.

Additionally, our Western blot results are consistent with the whole-cell patch-clamp recordings. When blotting for hERG protein, appearance of a 155-kDa protein band is associated with normal hERG processing and is a marker for pharmacological rescue [4], [6], [22]. Our study shows that E637K-hERG cells expressed almost entirely as the 135 kDa band after treatment with either PD-118057 or thapsigargin. Similarly, cells expressing WT/E637K-hERG were not rescued with either drug treatment. Taken together, our findings show that both PD-118057 and thapsigargin enhance WT-hERG channel current, but neither drug affects the current magnitude, gating kinetics or defective protein trafficking of the dominant negative WT/E637K-hERG mutant. Although not directly clinically applicable, these may impact the development of new therapies for diseases caused by the dominant-negative effect of LQT2 mutations. More importantly, experiments in animals or isolated hearts are needed to assess the potential therapeutic value and safety profile of PD-118057 and thapsigargin.

## Supporting Information

Table S1 **Candidate pharmacologic agents for the rescue of hERG current and their proposed mechanisms of action.** High variability exists in both the potential binding sites and resultant bioelectric alterations.
(DOC)
