# Supplementary material for: Pharmacologic Approach to Defective Protein Trafficking in the E637K-hERG Mutant with PD-118057 and Thapsigargin
Source: PLoS One. 2013 Jun 19;8(6):e65481. doi: 10.1371/journal.pone.0065481 (PMC3686757; doi:10.1371/journal.pone.0065481)
Supplement: Table S1 — Candidate pharmacologic agents for the rescue of hERG current and their proposed mechanisms of action. High variability exists in both the potential binding sites and resultant bioelectric alterations. (DOC) [file pone.0065481.s001.doc]

|  | **Type** | **Binding Site** | **Mechanism** | **Reference** |
| --- | --- | --- | --- | --- |
| **PRP260243** | Type 1 hERG channel activator | S5 or S6 domain | Enhance current by attenuating inactivation and slowing the rate of channel deactivation | 28,34 |
| **NS1643** | Type 2 hERG channel activator | the outer vestibule and pore entrance | Reduce channel inactivation | 29 |
| **PD-118057** | Type 2 hERG channel activator | the pore helix and a nearby region of S6 | Possibly bind to the channel directly and increase its open probability and activation potential | 27 |
| **Thapsigargin** | sarcoplasmic/endoplasmic reticulum calcium ATPase inhibitor | N/A | Block the ability of the cell to pump calcium into the sarcoplasmic/endoplasmic reticula | 13 |
